# Predicted Changes in Fatty Acid Intakes, Plasma Lipids, and Cardiovascular Disease Risk Following Replacement of *trans* Fatty Acid-Containing Soybean Oil with Application-Appropriate Alternatives

**DOI:** 10.1007/s11745-012-3705-y

**Published:** 2012-08-18

**Authors:** Michael Lefevre, Ronald P. Mensink, Penny M. Kris-Etherton, Barbara Petersen, Kim Smith, Brent D. Flickinger

**Affiliations:** 1 Department of Nutrition, Dietetics and Food Science, Utah State University, 9815 Old Main Hill, Logan, UT 84322-9815 USA; 2 Department of Human Biology, NUTRIM School for Nutrition, Toxicology and Metabolism, Maastricht University, Maastricht, The Netherlands; 3 Department of Nutritional Sciences, The Pennsylvania State University, University Park, PA 16802 USA; 4 Exponent Inc, Washington, DC 20036 USA; 5 Nutritional Science, Archer Daniels Midland Company, Randall Research Center, Decatur, IL 62521 USA

**Keywords:** *trans* fatty acid, Partially hydrogenated soybean oil (PHSBO), Cardiovascular disease, Linolenic acid

## Abstract

The varied functional requirements satisfied by *trans* fatty acid (TFA)—containing oils constrains the selection of alternative fats and oils for use as potential replacements in specific food applications. We aimed to model the effects of replacing TFA-containing partially hydrogenated soybean oil (PHSBO) with application-appropriate alternatives on population fatty acid intakes, plasma lipids, and cardiovascular disease (CVD) risk. Using the National Health and Nutrition Examination Survey 24-hour dietary recalls for 1999–2002, we selected 25 food categories, accounting for 86 % of soybean oil (SBO) and 79 % of TFA intake for replacement modeling. Before modeling, those in the middle quintile had a mean PHSBO TFA intake of 1.2 % of energy. PHSBO replacement in applications requiring thermal stability by either low-linolenic acid SBO or mid-oleic, low-linolenic acid SBO decreased TFA intake by 0.3 % of energy and predicted CVD risk by 0.7–0.8 %. PHSBO replacement in applications requiring functional properties with palm-based oils reduced TFA intake by 0.8 % of energy, increased palmitic acid intake by 1.0 % of energy, and reduced predicted CVD risk by 0.4 %, whereas replacement with fully hydrogenated interesterified SBO reduced TFA intake by 0.7 % of energy, increased stearic acid intake by 1.0 % of energy, and decreased predicted CVD risk by 1.2 %. PHSBO replacement in both thermal and functional applications reduced TFA intake by 1.0 % of energy and predicted CVD risk by 1.5 %. Based solely on changes in plasma lipids and lipoproteins, all PHSBO replacement models reduced estimated CVD risk, albeit less than previously reported using simpler replacement models.

## Introduction

On July 9, 2003, the US Food and Drug Administration (FDA) mandated that food manufacturers list the content of *trans* fatty acid (TFA) on the Nutrition Facts panel of foods and dietary supplements by January 1, 2006. Subsequently, additional legislation banning or restricting the use of fats and oils containing TFA has been passed in California as well as major US metropolitan areas such as New York City and Philadelphia, and has been considered in 23 other states [1]. These actions occurred in response to compelling evidence that dietary TFA increases the risk for cardiovascular disease (CVD) [2], most notably by raising total cholesterol (TC) and low-density lipoprotein cholesterol (LDL-C) while lowering high-density lipoprotein cholesterol (HDL-C) [3].

The food manufacturing and restaurant industries have responded by reformulating products and recipes to minimize their TFA content. Although the ensuing reduction in the population intake in TFA is predicted to reduce the risk for CVD, the extent of this risk reduction is dependent on the choice of TFA replacement fats. In 2005, prior to TFA labeling requirements, per capita availability of margarine and shortening totaled 33.1 lbs/year (15.05 kg/year) or 370 kcal/person/day [4]. Thus, the choice of alternative oils used to replace margarines and shortening has the potential to affect health by changing a substantial portion of the population’s fatty acid (FA) intake in addition to that of TFA alone.

The choice of TFA replacements within a given food is constrained by the desired functionality. TFA-containing fats and oils have two desirable properties: thermal stability for use in frying and an appropriate melting profile for use as shortening or margarine when solid fats are required. Consequently, a single replacement option for TFA is unlikely to satisfy the broad array of applications currently filled by TFA-containing oils. Furthermore, TFA replacement oils need to be available in sufficient amounts to meet domestic consumption demands, estimated in 2005 to be 8.6 × 10^9^ billion pounds (3.9 × 10^9^ kg) alone for soybean oil (SBO) used in baking, frying, and margarine applications [5].

As efforts to replace TFA-containing oils continue, guidance is needed with respect to which oils, or combination of oils, would likely provide the greatest improvements in CVD risk. In the present study, we model the effects of five application-appropriate TFA replacement scenarios on population intakes of FA. The choice of oils used in our replacement scenarios was based on both functional suitability and availability as TFA replacements. Issues of availability limited our choices to replacements based on either soy bean or palm oil. In food applications requiring oils with enhanced thermal stability (e.g., for fried foods) we selected two low linolenic acid soy bean oils alternatives: (1) a low linolenic acid soy bean oil in which the linolenic acid is reduced and replaced with linoleic acid; and (2) a low linolenic acid, mid oleic acid soybean oil in which both the linolenic and linoleic acid are reduced and replaced with oleic acid. In food applications requiring solid fats to provide functional properties (e.g., for pie crusts and margarines), we selected two solid fat alternatives with different fatty acid profiles: (1) palm oil in which the predominant saturated fatty acid is palmitic acid; and (2) interesterified, fully hydrogenated soybean oil in which the predominant saturated fatty acid is stearic acid. This modeling exercise enabled us to predict the effect of these replacement scenarios on reductions in CVD risk mediated by changes in plasma lipid/lipoprotein levels.

## Materials and Methods

### Modeling of Changes in Dietary FA Intake Following PHSBO Replacement

The methods for estimating SBO intake, TFA intake, and changes in dietary FA intakes following application-appropriate substitution of replacement oils and fats for PHSBO are essentially identical to those previously published [6, 7] and are detailed below.

#### Estimation of TFA Intake During 1999–2002

Dietary intake of whole foods was estimated using food consumption data from the 1999–2002 National Health and Nutrition Examination Survey (NHANES) [8], which included 9,965 participants in 1999–2000 and 11,039 participants in 2001–2002. All estimates were derived using the NHANES 4-year medical examination center statistical weights and adjusting for survey design.

NHANES researchers used two US Department of Agriculture (USDA) databases—the 1993 *trans* Fatty Acid Database [9] and the Nutrient Database for Standard Reference [10], in which many foods are the same—to map the TFA content of analyzed foods to the foods reported in the 1999–2002 NHANES. All analyses, including the total dietary TFA intakes, were calculated using the NHANES food consumption data and TFA content of individual foods using Foods Analysis and Residue Evaluation (FARE™) software (version 7.997; Exponent, Washington, DC). TFA consumption for individuals was calculated using the following formula:

$$ E_{\text{tfa}} = \sum\limits_{{i}} \left( {\text{Fc}} \right)_{i} \left( {{\text{TFA}}_{\text{f}} } \right)_{i} $$

where *E*_tfa_ is the total intake of TFA, *i* is the number of different food types consumed daily, Fc is the amount of food consumed (g/day), and TFA_f_ is the TFA content of food (g/100 g food).

#### Estimation of SBO Content of NHANES Foods

The SBO content of food (including both nonhydrogenated and PHSBO) was determined using the USDA-developed recipes that translate foods reported in the NHANES “as eaten” into their component ingredients (raw agricultural commodities) for purposes of nutrient analysis [8]. The recipes used in the FARE program are based on the USDA recipes but have been made more user friendly for use in additional kinds of intake analyses, including ingredients, additives, or contaminants. For example, the USDA recipes break foods down from the food reported as consumed (e.g., pizza) into the ingredients (e.g., dough, tomato sauce, and cheese). The final recipes were further broken down into the raw agricultural commodities (e.g., wheat flour, tomatoes, olive oil, milk-based fat, etc.). The final recipes have been quality checked and approved by the USDA and are currently used by the FDA.

The calculation for the SBO from foods reported consumed in the NHANES is similar to the formula presented earlier exception that the amount of food consumed was multiplied by the amount of SBO in food (derived from NHANES recipes) to estimate individual SBO intakes.

#### Selection of Food Categories for TFA Replacement Modeling

All NHANES foods were assigned to 1 of 59 broad food categories. These categories are based on the NHANES tiered food coding (i.e., all foods in the diet are grouped into three tiers starting with nine broad food categories and 263 additional subcategories). The selection of these categories was based on prior knowledge of their SBO content and degree of hydrogenation as communicated by the industry members of the ILSI North America Technical Committee on Dietary Lipids. The calculations for total dietary SBO and TFA intake within each food category were estimated as described above but on a food category basis. Based on these analyses, 25 food categories, accounting for 86 % of SBO intake and 79 % of TFA intake, were further considered for inclusion in our replacement models (Table 1).

**Table 1:** Fatty acid profiles of soybean oils assigned to broad food categories used for replacement models

Food categories	NHANES intake		Assigned soybean oil fatty acid composition (percentage of total fatty acids)						Assigned replacement fat
	TFA (g/day)	SBO (g/day)	16:0	18:0	18:1c	18:2n6	18:3n3	18:1t	
1. Frozen plate meals with grain mixture as major ingredient^a^	0.001	0.01	10.5	6.5	26.3	35.0	3.6	12.7	F1A; F2A
2. Squeeze or liquid butters^a^	0.003	0.02	10.1	3.6	21.2	51.3	6.8	0.7	Not replaced
3. Frozen or shelf-stable plate meals with meat, poultry, fish as major ingredient^a^	0.006	0.02	11.5	6.0	24.7	34.6	3.0	6.6	F1A; F2A
4. Cooking fats (shortening)^a^	0.006	0.15	10.5	6.5	26.3	35	3.6	12.7	F1A; F2A
5. Salad dressings, oil based^2a^	0.007	0.63	10.1	3.6	21.2	51.3	6.8	0.7	Not replaced
6. Soups, creamed based^a^	0.011	0.06	11.1	9.3	30.2	15.0	0.8	23.1	F1A; F2A
7. French fries, baked from frozen^a^	0.015	0.02	11.5	10.4	28.3	6.2	0.3	34.2	H1; H2
8. Soups, broth based^a^	0.018	0.12	10.1	3.6	21.2	51.3	6.8	0.7	Not replaced
9. Mayonnaise^a^	0.024	1.18	10.1	3.6	21.2	51.3	6.8	0.7	Not replaced
10. Pretzels and other grain snacks^a^	0.036	0.06	11.1	9.3	30.2	15.0	0.8	23.1	F1A; F2A
11. White potatoes, chips and sticks^a^	0.069	0.13	11.1	9.3	30.2	15.0	0.8	23.1	H1; H2
12a. Fried fish including breaded and fried, commercially fried^a^	0.080	0.41	11.1	9.3	30.2	15.0	0.8	23.1	H1; H2
12b. Fried fish including breaded and fried, home fried^b^	Included in 12a		10.4	5.5	24.1	39.3	4.8	8.1	H1; H2
13a. Fried poultry including breaded and fried, commercially fried^a^	0.092	0.43	11.1	9.3	30.2	15.0	0.8	23.1	H1; H2
13b. Fried poultry including breaded and fried, home fried^b^	Included in 13a		10.4	5.5	24.1	39.3	4.8	8.1	H1; H2
14a. Fried beef, veal, and pork, including breaded and fried, commercially fried^a^	0.097	0.12	11.1	9.3	30.2	15.0	0.8	23.1	H1; H2
14b. Fried beef, veal, and pork, including breaded and fried, home fried^b^	Included in 14a		10.4	5.5	24.1	39.3	4.8	8.1	H1; H2
15a. Fried eggs, commercially fried^a^	0.110	0.53	11.1	9.3	30.2	15.0	0.8	23.1	H1; H2
15b. Fried eggs, home fried^b^	Included in 15a		10.4	5.5	24.1	39.3	4.8	8.1	H1; H2
16. Tub margarine^b^	0.122	0.60	17.9	9.2	24.8	17.8	2.5	11.7	F1B; F2B
17. Salad dressings, cream based^a^	0.126	1.36	10.1	3.6	21.2	51.3	6.8	0.7	Not replaced
18. Stick margarine^b^	0.174	0.66	13.7	7.9	22.4	19.4	2.2	18.7	F1C; F2C
19. Popcorn^b^	0.207	0.43	11.1	13.5	22.0	4.0	0.2	43.3	F1A; F2A
20. Crackers^a^	0.215	0.63	11.1	9.3	30.2	15.0	0.8	23.1	F1A; F2A
21. Tortilla chips and other corn-based snacks^a^	0.333	1.05	10.8	7.9	28.3	25.0	2.2	17.9	H1; H2
22. Grain mixtures, ethnic dishes (Mexican, Puerto Rican, Asian, Italian)^a^	0.565	2.82	11.1	9.3	30.2	15.0	0.8	23.1	F1A; F2A
23. French fries, commercial^b^	0.643	2.02	11.2	9.7	29.6	12.1	0.6	26.7	H1; H2
24a. Yeast bread, including breaded non-fried meats, excluding sweet rolls and biscuits^b^	0.708	4.18	10.2	4.4	22.4	47.2	6.0	3.7	Not replaced
24b. Sweet rolls and biscuits^b^	Included in 23a		11.3	9.9	29.3	10.6	0.6	28.6	F1A; F2A
25. Cakes, cookies, pies, pastries, pancakes, waffles, French toast^a^	1.125	4.02	11.1	9.3	30.2	15.0	0.8	23.1	F1A; F2A
Sum of 25 categories	4.8	21.7							
Total diet	6.1	25.2							
25 categories as % of total diet	79 %	86 %							

NHANES, National Health and Nutrition Examination Survey; SBO, soybean oil; TFA, *trans* fatty acid. For replacement fats: *F1A* functional model 1A, *F2A* functional model 2A, *F1B* functional model 1B, *F2B* functional model 2B, *F1C* functional model 1C, *F2C* functional model 2C, *H1* heat-stable model 1, *H2* heat-stable model 2

^a^Fatty acid composition for the PHSBO used in the products derived from the USDA *Trans* Fatty Acid Database [9]

^b^Fatty acid composition for the PHSBO used in the products assigned by ILSI Technical Committee on Fatty Acids based on member companies’ estimate of product composition in the marketplace between 1999 and 2002

#### Reference Consumption of FA from SBO

For the purposes of this analysis, we defined the consumption of FA from the SBO fraction of foods during the 1999–2002 NHANES survey period as our “reference.” This period was selected because it immediately preceded significant efforts by the food industry to remove TFA from products.

The NHANES database does not include the FA composition of the individual components (e.g., SBO) of foods. The SBO used in foods is either liquid (nonhydrogenated) or partially hydrogenated to a particular level. The FA composition of the SBO will vary depending on the level of hydrogenation. Because it would be impossible to make assumptions regarding the specific TFA composition of the oils used for several thousand foods containing SBO, we simplified our analysis by assigning a SBO with a specified degree of hydrogenation and TFA content to each of the targeted 25 broad food categories. We then used the category-assigned SBO composition to estimate the SBO FA composition for all individual foods within each category.

The 1993 USDA *trans* Fatty Acid Database [9] and the USDA Nutrient Database for Standard Reference (Release 15) [10] were used to assign the FA content (including TFA) of SBO to each of the selected 25 broad food categories. The USDA *trans* Fatty Acid Database has FA profiles for the oil in 214 foods. When this database did not provide the FA profiles for specific varieties of SBO used in target food groups, these FA were estimated using information from the food industry (ILSI North America Technical Committee on Dietary Lipids). The FA data for SBO within the 25 broad food categories were chosen to reflect what was in the food marketplace during the 1999–2002 NHANES survey years (Table 1).

When a specific oil was not listed in a food recipe for oils, margarines, or shortenings, a Monte Carlo approach was used to select an oil for this food. Oil varieties used in the analysis were based on the USDA oil production statistics [11]. The SBO used to estimate reference FA intakes ranged in TFA content from 0.7 % of FA (liquid SBO, not hydrogenated) to 43.3 % of FA (for popcorn) (Table 1).

Individual SBO TFA intakes from both nonhydrogenated and partially hydrogenated SBO (henceforth combined and collectively referred to as PHSBO) were determined and the population was subsequently grouped by quintiles of PHSBO TFA intake. Once an NHANES respondent was assigned to a particular quintile in the reference analysis, we computed the mean intakes of the major FA within each quintile of PHSBO TFA intake. Respondents remained in the same population quintile for all subsequent replacement model analyses.

#### Consumption of FA Following PHSBO Oil Replacement

To determine the dietary impact of substituting new oils for existing partially PHSBO in the diet, five application-appropriate replacement models were considered.

*Heat*-*Stable Model 1* A nonhydrogenated low-linolenic acid SBO was substituted in applications requiring thermal stability (e.g., frying). Foods included were tortilla chips and other corn-based baked snacks; commercially and home fried meat, fish, poultry, eggs, and French fries; home-baked French fries; and potato chips.

*Heat*-*Stable Model 2* A nonhydrogenated mid-oleic, low-linolenic acid SBO was substituted in the same foods as in heat-stable model 1.

*Functional Model 1* Palm-based oils were substituted in applications requiring specific functional characteristics (e.g., melting profile and shortening applications). Foods included were stick and tub margarines; shortening; baked goods; crackers, pretzels, and other grain snacks; creamy soups; grain mixed dishes; sweet rolls and biscuits; popcorn; and frozen meals.

*Functional Model 2* Fully hydrogenated interesterified (FH-IE)–based SBO, high in stearic acid, were substituted in the same foods as in functional model 1.

*Complete Model* A 50:50 ratio of the oils used in the heat-stable models 1 and 2 was substituted in thermal applications and a 50:50 ratio of the oils used in functional models 1 and 2 was substituted in functional applications.

To simplify our analyses, only the PHSBO portion of the food within the 25 food categories was subject to replacement modeling. The FA composition of the oils used in the replacement models, along with that of nonhydrogenated SBO and a typical PHSBO, are presented in Table 2. A review of the TFA content of the 25 categories revealed a number of categories that would have had a low degree of SBO hydrogenation between 1999 and 2002 [12]. These categories included “squeeze or liquid butters,” “soups, broth based,” “salad dressing, oil based,” “salad dressing, creamed based,” “mayonnaise,” and “yeast breads—other than sweet rolls and biscuits” (Table 1). Consequently, the reference FA composition of these categories was retained throughout all replacement scenarios.

**Table 2:** Nutrient profiles of reference SBO and oils used in replacement models

Replacement oil	Application	C16:0	C18:0	C18:1c	C18:2n6	C18:3n3	C18:1t
Reference: soybean oil	Frying	10.5	4.4	22.6	50.4	6.8	0.0
Reference: typical PHSBO	Frying and shortening	11.1	9.3	30.2	15.0	0.8	23.1
Heat-stable 1: low-linolenic acid, SBO based	Frying	10.5	4.4	25.9	56.1	2.0	0.7
Heat-stable 2: mid-oleic, low-linolenic acid SBO	Frying	9.5	3.5	50.0	35.0	2.8	0.7
Functional 1A: palm oil based	Shortening	42.0	4.0	40.5	10.0	0.0	1.0
Functional 2A: FH-IE SBO based	Shortening	11.5	35.1	15.2	30.4	2.5	3.9
Functional 1B: palm oil based	Tub margarine	18.1	3.7	23.9	37.3	4.8	0.8
Functional 2B FH-IE SBO based	Tub margarine	10.3	11.0	21.3	46.3	5.9	1.5
Functional 1C: palm oil based	Stick margarine	24.3	2.8	40.2	11.7	3.7	0.9
Functional 2C: FH-IE SBO based	Stick margarine	9.1	17.8	26.5	35.5	5.5	2.2

Data are the percentages of total fatty acid

FH-IE, fully hydrogenated interesterified; PHSBO, partially hydrogenated soybean oil; SBO, soybean oil

Once the PHSBO replacements were made, we computed the predicted mean intakes of each of the FA within each quintile of reference PHSBO TFA intake.

#### Calculation of 20-Year CVD Risk

Predicted changes in FA intake from reference values were estimated within each PHSBO TFA quintile for each of the five replacement models. Using published regression equations [3], we calculated the predicted mean change in TC, LDL-C, HDL-C, triglycerides, and the TC/HDL-C ratio based on dietary FA replacements. From the TC/HDL-C ratio, we estimated the 20-year risk for developing CVD from equations provided by Anderson et al. [13]. Predicted change in relative risk was defined as follows: (model 20-year CVD risk)/(reference 20-year CVD risk)—1.

#### Data Management and Statistical Analyses

All NHANES data management, TFA replacement modeling, and estimates of means, medians, and percentile distributions for dietary FA intakes under each replacement model were accomplished using FARE software. Estimates of changes in lipid levels and coronary heart disease risk associated for each replacement model were calculated using an Excel (Microsoft, Redmond, WA) spreadsheet populated with the relevant equations and coefficients.

## Results

### Population TFA Intake

Based on the USDA *trans* Fatty Acid Database and the USDA Nutrient Database for Standard Reference, the mean population total TFA intake during 1999–2002 was 2.5 % of energy with a 90th percentile intake at 4.3 % of energy (Table 3). Those in the highest quintile of TFA intake had a mean total TFA intake of 4.5 % of energy with a range between 3.5 and 12.5 % of energy.

**Table 3:** Estimated and modeled per capita intake of TFA for the U.S. population (aged ≥3 years) as a percentage of total energy

Replacement model	Mean	90th percentile	TFA intake quintile^a^				
			1	2	3	4	5
TFA intake from NHANES 1999–2002 data	2.5	4.3	0.9 (0–1.3)	1.7 (1.3–2.0)	2.3 (2.0–2.6)	3.0 (2.6–3.5)	4.5 (3.5–12.5)
Reference TFA intake from PHSBO	1.4	3.0	0.2 (0–0.4)	0.7 (0.4–0.9)	1.2 (0.9–1.5)	1.9 (1.5–2.3)	3.2 (2.3–10.5)
Heat-stable 1: low-linolenic SBO	1.1	2.5	0.1	0.5	0.9	1.4	2.5
Heat-stable 2: mid-oleic, low-linolenic SBO	1.1	2.5	0.1	0.5	0.9	1.4	2.5
Functional 1: palm-based oil	0.5	1.3	0.1	0.3	0.4	0.6	0.9
Functional 2: FH-IE SBO	0.6	1.4	0.1	0.3	0.5	0.8	1.2
Combined: both heat-stable and functional replacements	0.2	0.4	0.1	0.2	0.2	0.2	0.3

FH-IE, fully hydrogenated interesterified; PHSBO, partially hydrogenated soybean oil; SBO, soybean oil; TFA, *trans* fatty acid

^a^For quintiles, data are mean (range)

Estimated mean TFA intake from PHSBO in the top 25 SBO-containing food categories was 1.4 % of energy, accounting for 56 % of the value derived for the population total TFA intake (Table 3). The 90th percentile for PHSBO TFA was 3.0 % of energy (70 % of the value derived for the population total TFA intake). Taking into account naturally occurring TFA from beef and dairy products (estimated at 21 % of total TFA intake) [10], the estimated mean TFA intake from PHSBO in the top 25 SBO-containing food categories accounted for 71 % of the population total TFA intake from hydrogenated vegetable oils.

### Predicted Effects of Application-Appropriate PHSBO Replacement on Product FA Composition

**Fig. 1:**
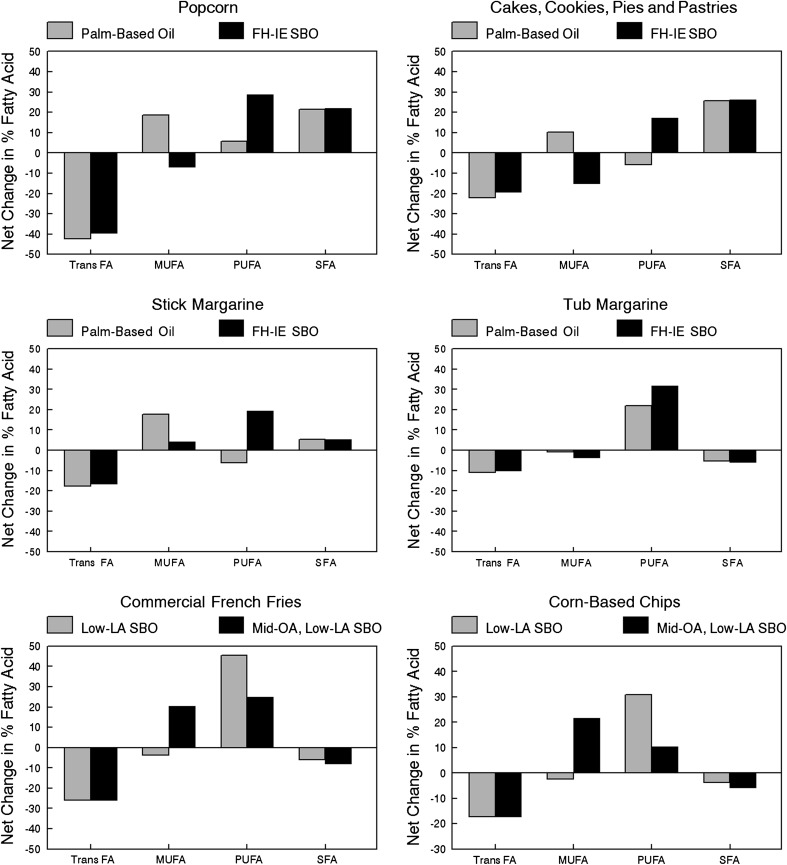
Predicted change in fatty acid content of selected food categories with replacement of partially hydrogenated soybean oil (PHSBO) with application-appropriate oils. Graphs show the predicted net changes (as a percentage of total fatty acids) in *trans* fatty acid (TFA), MUFA, PUFA, and SFA following replacement of PHSBO with either palm-based oils of fully hydrogenated interesterified soybean oil in foods requiring a functional fat (popcorn, cakes, cookies, pies, pastries, stick margarine, tube margarine) or with low-linolenic acid soybean oil or mid-oleic, low-linolenic acid soybean oil in foods requiring heat-stable oils (commercial French fries, corn-based chips)

The predicted effects of the replacement of PHSBO with application-appropriate oils on FA classes for selected food product groups are shown in Fig. 1. The food product groups in Fig. 1 were selected to indicate a representative range of predicted changes in FA composition. Net predicted change in TFA content (as a percentage of total FA) ranged from a net reduction of approximately 10 % of FA for tub margarine to >40 % of FA for popcorn with most other products ranging between 20 and 25 % of FA. For popcorn, cakes, cookies, pies, and pastries that require PHSBO for functional properties, replacement of PHSBO with either palm-based oils or FH-IE SBO increased predicted total saturated FA (SFA) by 20–25 % of FA. In contrast, replacement of PHSBO with either palm-based oils or FH-IE SBO in stick margarine is predicted to result in a moderate net increase in SFA by approximately 5 % of FA, whereas in tub margarines SFA are predicted to decrease by approximately 5 % of FA.

For products in which PHSBO is used for thermal stability (French fries, corn chips) replacement of PHSBO with low-linolenic acid SBO (heat-stable model 1) increased predicted PUFA levels between 30 and 45 % of FA, whereas predicted *cis*-MUFA levels were largely unchanged. In the same applications, replacement of PHSBO with mid-oleic, low-linolenic acid SBO (heat-stable model 2) increased predicted PUFA levels by 10–25 % of FA, whereas predicted MUFA levels increased by approximately 20 %.

### Effect of Application-Appropriate PHSBO Replacement on Population FA Intakes

Replacement of PHSBO in applications requiring thermal stability with either low-linolenic acid SBO (heat-stable model 1) or mid-oleic, low-linolenic acid SBO (heat-stable model 2) produced identical predicted reductions in mean PHSBO TFA intakes of 0.3 % of energy (25 % decrease) in the middle and 0.7 % of energy (22 % decrease) in the upper quintile of PHSBO TFA intake (Table 3). Both heat-stable replacement models increased predicted linoleic acid intake, whereas the use of mid-oleic, low-linolenic SBO predictably increased oleic acid intake. Modest reductions in total SFA were predicted in the high TFA consumers, whereas linolenic acid was not predicted to change with either heat-stable replacement oil (Table 4).

**Table 4:** Estimated mean fatty acid intakes from SBO (including PHSBO) at reference and under five application-appropriate replacement models for the middle and upper population quintiles for PHSBO TFA intake

Replacement model	Population quintile	C16:0	C18:0	C18:1c	C18:2n6	C18:3n3	Total SFA (C14:0-C18:0)
Reference	3rd	1.0	0.6	2.4	3.1	0.4	1.6
Heat-stable 1: low-linolenic SBO	3rd	1.0	0.6	2.4	3.6	0.4	1.6
Heat-stable 2: mid-oleic; low-linolenic SBO	3rd	1.0	0.5	2.7	3.3	0.4	1.5
Functional 1: palm-based oil	3rd	2.0	0.5	2.8	2.9	0.3	2.5
Functional 2: FH-IE SBO	3rd	1.0	1.5	2.0	3.7	0.4	2.5
Combined: both heat-stable and functional replacements	3rd	1.5	0.9	2.5	3.7	0.4	2.4
Reference	5th	1.8	1.4	4.7	4.0	0.4	3.2
Heat-stable 1: low linolenic SBO	5th	1.8	1.2	4.5	5.3	0.4	3.0
Heat-stable 2: mid-oleic; low linolenic SBO	5th	1.8	1.2	5.3	4.6	0.5	3.0
Functional 1: palm oil based	5th	4.8	0.9	5.9	3.7	0.3	5.7
Functional 2: FH-IE SBO	5th	1.9	3.8	3.5	5.7	0.6	5.7
Combined: both heat-stable and functional replacements	5th	3.2	2.1	4.8	5.6	0.5	5.3

Data are for the US population (aged ≥3 years) as a percentage of total energy

FH-IE, fully hydrogenated interesterified; PHSBO, partially hydrogenated soybean oil; SBO, soybean oil; SFA, saturated fatty acid; TFA, *trans* fatty acid

Replacement of PHSBO used in functional applications with palm-based oils reduced predicted PHSBO TFA intake by 0.8 % of energy (67 % decrease) in the middle and 2.3 % of energy (72 % decrease) in the upper quintiles of PHSBO TFA intake (Table 3). These predicted reductions in PHSBO TFA were accompanied by 1.0 and 3.0 % predicted increases in energy from palmitic acid in the middle and upper PHSBO TFA intake quintiles (Table 4). Use of palm-based oils also produced slight predicted reductions in linoleic and linolenic acid and increases in oleic acid.

Because of its residual TFA content (as provided for commercial food ingredient products at the time), replacement of PHSBO used in functional applications with FH-IE SBO produced smaller predicted reductions in PHSBO TFA intakes than observed with palm-based oil. Replacement of PHSBO with FH-IE SBO reduced predicted mean PHSBO TFA intake by 0.7 % of energy (58 % decrease) in the middle and 2.0 % of energy (63 % decrease) in the upper quintiles of SBO TFA intake (Table 3). These predicted reductions in SBO TFA were accompanied by 0.9 and 2.4 % predicted increases in energy intake from stearic acid in the middle and upper quintiles, whereas predicted changes in total SFA were identical to those observed in the palm-based oil replacement model (Table 4). Use of FH-IE SBO also produced predicted reductions in oleic acid and increases in linoleic acid intakes.

Combined replacement of PHSBO used in both thermal and functional applications with equal contributions from each application-appropriate oil produced the greatest predicted reduction in PHSBO TFA intake. Predicted PHSBO TFA intake decreased by 83 % (1.0 % of energy) in the middle quintile, whereas predicted intake decreased by 91 % (2.9 % of energy) in the upper quintile (Table 3). These predicted changes in PHSBO TFA intakes were accompanied by predicted increases in palmitic, stearic, and linoleic acids with virtually no predicted change in either oleic or linolenic acids (Table 4).

### Effect of Application-Specific PHSBO Replacement on Estimated 20-Year CVD Risk

**Fig. 2:**
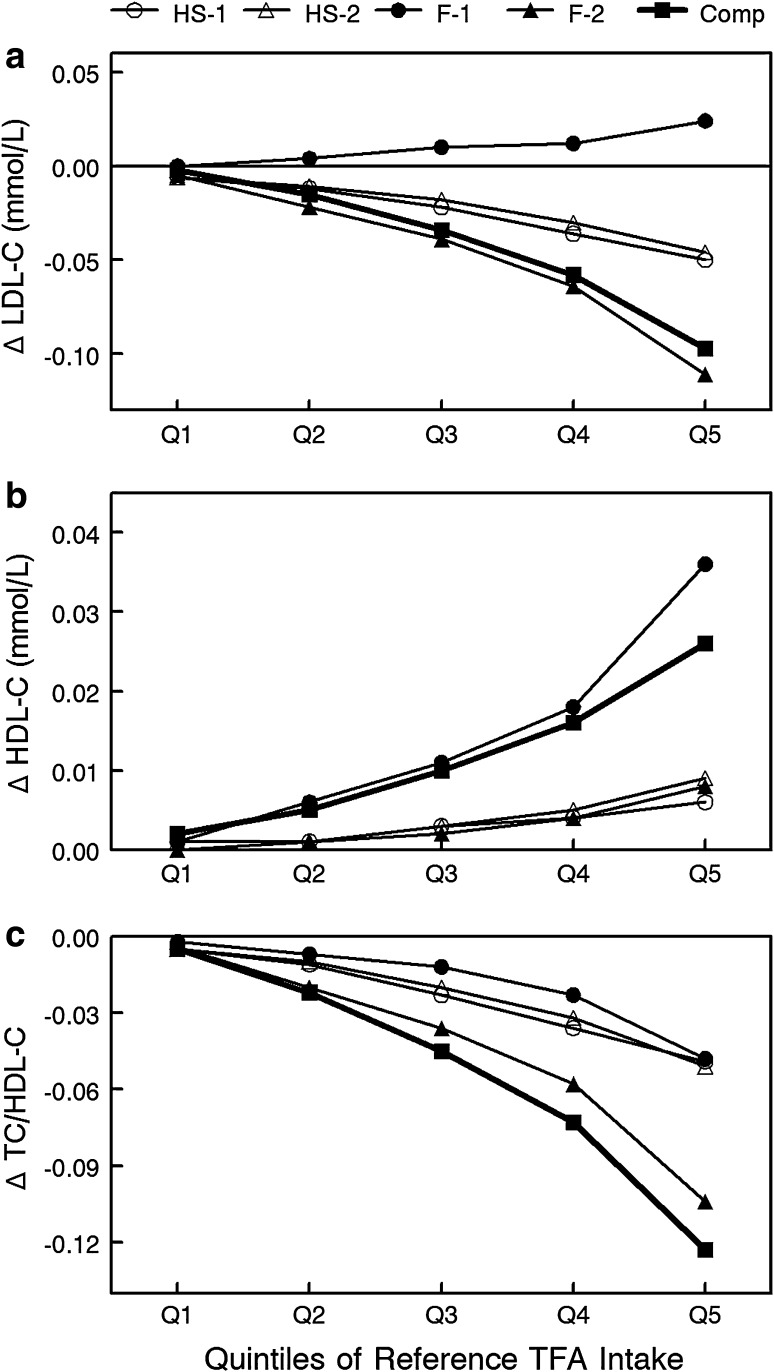
Predicted change in **a** low-density lipoprotein cholesterol, **b** high-density lipoprotein cholesterol, and **c** total cholesterol/high-density lipoprotein cholesterol ratio for each quintile of reference *trans* fatty acid intake. HS-1, heat-stable model 1 using nonhydrogenated low-linolenic acid soybean oil (SBO) as a replacement in applications requiring thermal stability; HS-2, heat-stable model 2 using nonhydrogenated mid-oleic, low-linolenic acid SBO as a replacement in applications requiring thermal stability; F-1, functional model 1 using palm-based oils in applications requiring specific functional characteristics; F-2, functional model 2 using fully hydrogenated interesterified-based SBO in applications requiring specific functional characteristics; Comp, complete replacement using a 50:50 ratio of the oils used in heat-stable models 1 and 2 and a 50:50 ratio of the oils used in functional models 1 and 2 in their appropriate applications

**Fig. 3:**
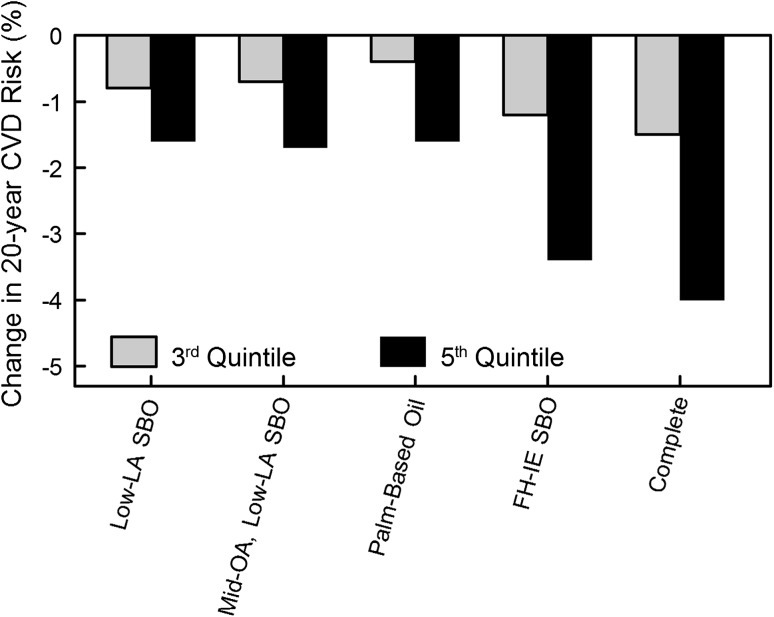
Predicted change in 20-year cardiovascular disease (CVD) risk following near complete replacement of partially hydrogenated soybean oil (PHSBO) with application-appropriate oils across quintiles of reference soybean oil *trans* fatty acid intake. Reference risk assumed a 40-year-old man without diabetes, hypertension, or left ventricular hypertrophy with a total cholesterol (TC) level of 200 mg/dL and an high-density lipoprotein cholesterol (HDL-C) level of 45 mg/dL. Changes in risk are based on predicted changes in the TC/HDL-cholesterol ratio subsequent to changes in predicted fatty acid intake following PHSBO replacement. *Low-LA SBO* low-linolenic acid soybean oil, *Mid-OA, Low-LA SBO* mid-oleic, low-linolenic acid soybean oil, *FH-IE SBO* fully hydrogenated interesterified soybean oil; complete, for heat-stable applications: 50:50 use of low-linolenic acid soybean oil and mid-oleic, low-linolenic acid soybean oil and for functional applications: 50:50 use of palm-based oils and fully hydrogenated interesterified soybean oil

From predicted changes in FA intakes relative to reference values, we calculated predicted changes in TC, LDL-C, HDL-C, and the TC/HDL-C ratio. With the exception of replacement with palm-based oil, LDL-C was predicted to decrease in all models (Fig. 2a). All replacement models showed a predicted increase in HDL-C (Fig. 2b) and decrease in the TC/HDL-C ratio (Fig. 2c). Predicted 20-year CVD risk (derived from changes in TC/HDL-C) declined with each of the five replacement models (Fig. 3). Predicted changes in CVD risk were similar with both heat-stable replacement models with risk declining by 0.7–0.8 % in the middle and 1.6–1.7 % in the upper quintiles of SBO TFA intake. In contrast, estimated CVD reduction differed between the two functional replacement models. Use of a palm-based oil reduced predicted CVD risk by 0.4 % in middle and by 1.6 % in the upper quintiles. In contrast, the FH-IE SBO reduced predicted CVD risk by 1.2 % in the middle and 3.4 % in the upper quintiles. The complete replacement model reduced predicted CVD risk by 1.5 % in the middle and 4.0 % in the upper quintiles.

## Discussion

Our study shows for the first time the predicted changes in the population intake of dietary FA associated with achievable application-appropriate replacements for PHSBO. With near complete replacement of PHSBO used in thermal and functional applications with an equal mix of likely available options, we predicted that TFA intake would decline by >90 % (2.9 % of energy) in the upper quintile of TFA users with increases in palmitic acid (1.4 % of energy), stearic acid (0.7 % of energy), and linoleic acid (1.6 % of energy) intakes and no appreciable change in oleic or linolenic acid intakes. Based exclusively on predicted changes in TC/HDL-C levels, we estimated that these changes in dietary FA would result in a 20-year CVD risk reduction of 1.5 % in the middle and 4.0 % in upper TFA quintiles.

Our choice of using TC/HDL-C as our primary metric is justified based on observations that unlike saturated FA, TFA both increase LDL-C and decrease HDL-C, resulting in uniquely unfavorable increases in the TC/HDL-C [3]. Indeed, the ability of TFA to increase TC/HDL-C has been used as a primary rationale in the call for its reduction in the diet [14]. The use of the TC/HDL-C ratio as the primary metric to evaluate changes in CVD risk associated with replacement of TFA in the diet is also supported in the World Health Organization scientific update on TFA [15].

The time period used for our reference analysis (1999–2002 NHANES survey), coupled with industry data for TFA content of foods in the marketplace at that time, allowed us to estimate the effects of TFA replacement prior to significant product reformulation. Our estimate of a mean total TFA intake of 2.5 % of energy agrees with a previous estimate of 2.6 % of energy from the Continuing Survey of Food Intakes of Individuals data for 1989–1991 [16] and 1994–1996 [12].

The mean SBO TFA content during the reference time period for the foods used in our replacement model was 56 % of that derived from the NHANES data, a difference due to several factors. To simplify our analysis, we focused only on SBO as the TFA source. Thus, partially hydrogenated oils from other sources (e.g., corn, canola, and cottonseed), which may contribute up to 18 % of partially hydrogenated oil consumption [17], were not considered in our analysis. Second, in selecting only the 25 SBO-containing food categories for analysis, we largely excluded TFA from animal sources, which may account for 21 % of TFA intake [12]. Finally, we updated the TFA content of selected foods, including items such as creamy salad dressings and sauces in which the TFA content reported in the USDA *trans* Fatty Acid Database was likely to be substantially higher than what was present in the marketplace during 1999–2002 [12].

Changes in selected food product FA composition in the United States between 2005 and 2008 resulting from replacement of TFA have been reported [18]. Analysis of changes in microwave popcorn FA composition indicated a net reduction of approximately 40 % in TFA and a net increase of 24 % in SFA. This compares favorably with our estimates of a net reduction of 40–42 % in TFA and a net increase of 22 % in SFA. For cakes and cookies, our predicted changes in TFA and SFA are approximately half of that reported to have occurred (estimated 40 % net reduction in TFA and 60 % net increase in SFA for the United States). These differences could reflect the selection of products higher in TFA for longitudinal FA analysis as opposed to the broader spectrum of foods within the cakes and cookies categories used in our estimates. Finally, our predicted changes in the product categories also are consistent with reported reductions in TFA + SFA and increases in *cis*-unsaturated FA following product reformulation in Canada [19]. With the exception of a predicted modest 3–7 % net increase in TFA + SFA levels in the cakes, cookies, pies, and pastries category, predictions for all other food categories indicated a net decrease in the TFA and SFA levels and a net increase in the *cis*-unsaturated FA levels (data not shown).

Our analysis accounted for approximately 71 % of TFA from vegetable sources. The remaining 29 % likely comes from hydrogenated corn, canola, and cottonseed oil not included in our replacement model. Assuming that these were similarly subjected to replacement would further decrease predicted CVD risk by an additional 40 % relative to our original estimates resulting in a 20-year CVD risk reduction of 2.1 % in the middle and 5.6 % in the upper TFA quintiles. However, even after corrections for unaccounted TFA from vegetable sources, our estimate for CVD risk reduction based on changes in the TC/HDLC ratio is less than half of that predicted by others (estimated at 6 % for the population average) [2]. Most of this difference can be traced to the choice of specific replacement oils. Earlier studies [2, 20] modeled the health effects of TFA replacement using simpler approaches in which all dietary TFA are replaced with a single class of FA (MUFA, PUFA, or SFA). Although this approach has been useful in providing guidance about the preferred FA to replace TFA to achieve maximum CVD risk reduction, it fails to consider certain practical issues for commercial food production.

Our approach was fundamentally different from previous studies in three respects. First, to reflect the food reformulation process more accurately, the unit of exchange in our analysis was whole oils and not individual FA. This approach adds complexities by taking into consideration changes in FA beyond TFA in terms of what is removed with the TFA-containing oil and what is added with the replacement oil.

Second, our approach considers the functional attributes required of the replacement oil to effectively substitute for TFA-containing oils within a given broad application. Thus, although oils high in PUFA or MUFA may be considered suitable replacements for many frying applications (accounting for approximately 25 % of TFA intake), they would not provide the desired functional characteristics in many baking applications.

Finally, because of the magnitude of TFA usage, particularly as PHSBO, the options for oil replacement are limited by availability and economics. Although both corn and sunflower oil have been used to replace TFA-containing oils for frying applications, their combined domestic production in 2006 was only 15 % of that of SBO [21] and supply reliability for commercial use is questionable [17]. The emergence of trait-enhanced SBO low in linolenic acid with or without increased oleic acid levels [17] suggests that they will likely constitute a substantial fraction of the oils used to replace TFA in frying applications. For functional applications, palm-based oil replacements have been used in Europe for over a decade and are currently available in sufficient quantities to meet demand [22]. However, because of the magnitude of domestic SBO production, we included FH-IE SBO in our models because it is likely that functional limitations with FH-IE SBO will shortly be overcome and it will be available as an alternative to palm oil.

Our estimates for CVD risk reduction are based only on the predicted effects of changes in individual FA on circulating lipoproteins. Our models do not consider possible effects on other CVD risk factors including inflammation and hemostatic factors, lipoprotein[a], and endothelial function, all of which are affected by TFA and other FA [2]. Thus, the actual reduction in CVD risk associated with TFA replacements may be greater than our estimates. Indeed, employing simpler substitution models, the estimates for CVD risk reduction based on data from prospective studies have been approximately four times higher than that predicted from changes in the TC/HDL-C ratio [2]. Based on changes in FA classes (and not individual FA) and published regression coefficients from prospective studies [23], our data would predict a reduction in CVD events of 11 % in the middle and 29 % in the upper quintiles of TFA users under the complete replacement model.

It is important to note that each of the proposed replacement strategies has uncertainties. The use of less thermally stable frying oils may result in increased consumption of oxidized and thermally degraded FA with potential adverse health effects [24]. Expanded use of low-linolenic SBO beyond that needed for applications requiring thermal stability (e.g., salad and cooking oils) may decrease population intakes of this beneficial n3-FA. Although oils high in oleic acid are generally considered to be healthy, some have cautioned against their increased consumption because of a similar atherogenic potential when compared to saturated fat in animal models [25], and recent data suggest that MUFA may actually increase coronary heart disease risk relative to SFA [26]. As a TFA replacement for functional applications, FH-IE SBO would seem to be preferred, largely because of the high content of cholesterol-neutral stearic acid. However, very high stearic acid (10.9 % of energy) intakes may increase fibrinogen levels [27]. Furthermore, the randomization of FA in the glycerol moiety of the triglyceride molecule by the interesterification process may adversely affect glucose metabolism at very high stearic acid intakes (12 % of energy) [28]. As a tropical oil, palm oil markedly increases LDL-C due to its high palmitic acid content [3]. These uncertainties warrant a continuing assessment of the health effects of new fats and oils introduced into the marketplace.

Finally, not considered in the current study was replacement of TFA-containing foods with other foods, rather than replacing their TFA-containing oils. Indeed, replacement of cakes, cookies, pies, French fries, chips, and other such foods with fruits, vegetables, and whole grains to achieve a dietary pattern consistent with current recommendations [29] would be expected to provide the greatest reduction in CVD risk.

In summary, all TFA replacement strategies evaluated changed the fatty acid profile in a manner that is projected to decrease CVD risk, albeit to different extents based on the specific selection of the replacement oils. The combined use of mid-oleic, low-linolenic SBO for frying applications and fully hydrogenated interesterified SBO for functional application is predicted to provide the greatest reduction in CVD risk. Nonetheless, our estimates of this CVD risk reduction, which are based solely on the predicted changes in plasma lipid profile, are lower than previous estimates due in large part to the real-world constraints associated with the selection of the TFA replacement oils. As more data are developed regarding the quantitative relationship between changes in dietary fatty acid composition and changes in a broader spectrum of CVD risk factors, the modeling employed in this study would be expected to provide even better estimates of the health consequences associated with population-wide changes in the use oils in the food supply. Such detailed modeling would be increasingly valuable as a tool to guide both policy makers and the food industry as further changes in the production, availability and use of alternate dietary oils are contemplated in efforts to provide a healthier food supply.

## Abbreviations

CVD: Cardiovascular disease
FA: Fatty acid
FARE: Foods Analysis and Residue Evaluation
FDA: US Food and Drug Administration
FH-IE: Fully hydrogenated interesterified
HDL-C: High-density lipoprotein cholesterol
LDL-C: Low-density lipoprotein cholesterol
NHANES: National Health and Nutrition Examination Survey
PHSBO: Partially hydrogenated soybean oil
SBO: Soybean oil
TC: Total cholesterol
TFA: *trans* fatty acid
USDA: US Department of Agriculture
